# Antidiabetic Effects of a Chinese Herbal Medicinal Compound Sangguayin Preparation via PI3K/Akt Signaling Pathway in* db/db* Mice

**DOI:** 10.1155/2018/2010423

**Published:** 2018-03-04

**Authors:** Qichang Xing, Yunzhong Chen

**Affiliations:** Hubei University of Chinese Medicine, Wuhan, Hubei 430065, China

## Abstract

Sangguayin (SGY), comprising four types of Chinese herbs, can be used as both food and medicine and has been clinically used to treat type 2 diabetes mellitus (T2DM) for a long time. Our previous study demonstrated the antidiabetic effect of SGY in experimental T2DM rats fed with a high-fat diet and treated with a low dose of streptozotocin. However, its mechanism of action is questionable. In this study, we refined the traditional SGY decoction and investigated its antidiabetic activity in* db/db* mice. We evaluated the possible molecular mechanism using skeletal muscle tissues. The results show that the treatment with SGY preparation resulted in a decrease in the blood glucose, glycated serum protein, and blood lipid levels and an improvement in the glucose tolerance as well as insulin resistance. In addition, SGY preparation remarkably upregulated the expression of insulin receptor, insulin receptor substrate-1, phosphoinositide 3 kinase (PI3K), protein kinase B (Akt), and glucose transporter type 4 (GLUT4). Thus, SGY preparation is an effective agent for the treatment of T2DM, and its molecular mechanism may be related to the regulation of PI3K/Akt signaling in the skeletal muscle.

## 1. Introduction

Type 2 diabetes mellitus (T2DM) is a metabolic disease caused by the impaired insulin production and/or decreased tissue response to insulin [1]. Given the high morbidity and mortality associated with T2DM, this disease is becoming a global health concern. The incidence of T2DM is expected to double (350 million) by the end of 2030 [2]. The pathogenesis of T2DM is unclear, but insulin resistance (IR) plays a major role in its occurrence and development. IR is characterized by impaired insulin regulation and metabolic function. Insulin-sensitive tissues fail to easily absorb glucose from the blood, resulting in high blood glucose levels in the body [3]. Several studies have demonstrated that the glucose metabolism in insulin-sensitive tissues is mainly mediated by the insulin receptor substrate- (IRS-) phosphoinositide 3 kinase- (PI3K-) protein kinase B (Akt) pathway [4]. These events lead to the transport of glucose transporter type 4 (GLUT4), which promotes the glucose uptake in skeletal muscles and ultimately lowers blood glucose levels [5]. Therefore, correcting IR in target tissues is an important treatment strategy for T2DM.

The existing synthetic antidiabetic drugs often exhibit side effects or resistance [6]. Hence, it is necessary to find better medicines from herbs or natural products. The traditional Chinese medicine (TCM), composed of several herbs, is often used as a prescription in clinical practice and known for controlling complex diseases such as diabetes. Several TCM prescriptions are proved to safely and effectively improve the lipid and glucose metabolism and used to treat T2DM [7]. Traditional Sangguayin (SGY), one of the series of Chinese prescriptions invented by Dr. Jiageng Li, comprises* Morus alba* L. (Moraceae),* Pueraria thomsonii* Benth (Leguminous),* Dioscoreae rhizoma *(Dioscoreaceae), and* Momordica charantia* L. (Cucurbitaceae) and has been reported to be clinically effective [8, 9]. We refined SGY by extracting total alkaloids, saponins, flavonoids, and polysaccharides from* M. alba*,* M. charantia*,* P. thomsonii*, and* D. rhizoma*, respectively, and formulated SGY preparation using these compounds in certain proportions. Furthermore, we analyzed its major chemical components with antidiabetic effects and the underlying molecular mechanisms using the* db/db* mouse. This mouse model presents syndromes, including hyperglycemia, obesity, and hyperlipidemia, while the disease pathogenesis and process are similar to human DM.

## 2. Materials and Methods

### 2.1. Sangguayin Preparation

The preparation of the effective components of the four herbs was performed as previously described and supplied by Key Laboratory of traditional Chinese medicine resources and traditional Chinese medicine compound. Alkaloids from* M. alba* were extracted by ethanol and purified with a cation-exchange resin. The total alkaloid content was over 70%. Saponins from* M. charantia *and flavonoids from* P. thomsonii* were obtained with ethanol extraction and macroporous resin absorption; the content of total saponins and flavonoid was more than 50%. Polysaccharides from* D. rhizoma* were extracted using water circumfluence and alcohol precipitation to obtain an extract with more than 50% total polysaccharides. All extracts were homogenously mixed at alkaloid/saponin/flavonoid/polysaccharide ratio of 1/6/5/6 (by weight). In our prior experiments, we have empirically determined the optimal hypoglycemic effect at this ratio.

### 2.2. Quality Control of SGY Preparation

The representative components from alkaloids, saponins, and flavonoids were individually analyzed for quality control.

The alkaloid extract of* M. alba* was subjected to precolumn derivatization with 2 g/L of 9-fluorenylmethyl chloroformate (Fmoc-Cl) and evaluated by DAD detector. ODS-C18 (4.6 mm × 250 mm, 5*μ*m) column was eluted with the mobile phase of acetonitrile and 0.2% phosphoric acid in a gradient mode at a flow rate of 1.0 mL/min. The detection wavelength was 263 nm. The content of 1-deoxynojirimycin (1-DNJ) was 15.08% that of the alkaloid extract.

The puerarin content of the flavonoid extract from* Pueraria *was determined by high-performance liquid chromatography (HPLC) using an ODS-C18 (4.6 mm × 250 mm, 5*μ*m) column. The mobile phase was methanol/0.1% acetic acid water (28 : 72, v/v), while the flow rate was 1.0 mL/min. The analysis was performed at 35°C and the absorbance was recorded at 245 nm wavelength. The content of puerarin was 30.4% that of the flavonoid extract.

The momordicoside A content of saponins from* M. charantia* extract was determined by HPLC analysis under the following conditions: TSKgel Amide-80 (4.6 mm × 250 mm, 5*μ*m) column; detection, evaporative light scattering detector (ELSD); drift tube temperature, 105°C; mobile phase, acetonitrile/water (70 : 30, v/v); gas flow rate, 2.7 L/min. The content of momordicoside A was 7.09% that of the saponins extracts.

We performed HPLC fingerprint analysis under the following conditions: TSKgel Amide-80 (4.6 mm × 250 mm, 5*μ*m) column; detection, ELSD; drift tube temperature, 90°C; mobile phase, acetonitrile/6.5 mM aqueous ammonium acetate in a gradient mode; gas flow rate, 2.7 L/min. SGY preparation samples were weighed and subjected to ultrasonic extraction with 50% methanol for 10 min. After centrifugation, the supernatant was filtered through a 0.45*μ*m membrane filter and 5*μ*L filtrate was injected into the HPLC column.

### 2.3. Animals

Male wild-type C57BLKS* (db/m)* mice and C57BLKS/*Lepr*db* (db/db)* mice (6-week old) were purchased from Nanjing Biomedical Research Institute of Nanjing University (NBRI; Nanjing, China). All animals were raised under the specific-pathogen-free environment with a standard light (12-h light/dark) and temperature condition (23 ± 2°C). The mice were fed with a standard chow diet and had free access to food and water. All experimental procedures were strictly performed as per the Guide of Care and Use of Laboratory and approved by the Ethics Committee of Hubei University of Chinese Medicine.

### 2.4. Methods

After 1 week of adaptive feeding, postprandial blood glucose (PBG) levels of* db/db* mice were determined with an Onetouch Ultra monitoring system. All* db/db* mice were randomly divided into four groups based on blood glucose levels such that the average blood glucose level was similar in each group. The control group was orally administered with normal saline, while the remaining three groups were daily orally administered with metformin at 250 mg/kg and SGY preparation at 175 and 700 mg/kg. The nondiabetic* db/m* mice were considered the normal control group. Body weight (BW) and PBG of mice were weekly monitored after the removal of food for 2 h [10]. Intraperitoneal glucose tolerance test (IPGTT) and intraperitoneal insulin tolerance test (IPITT) were conducted at weeks 7 and 8. After 9 weeks of intervention, mice were fasted for 12 h; the blood was taken by picking eyeball and immediately centrifuged (4°C, 1,500 ×g, 10 min) to obtain serum for biochemical analysis, while the skeletal muscles were treated with liquid nitrogen and stored at −80°C for western blot analysis.

### 2.5. Intraperitoneal Glucose Tolerance Test and Intraperitoneal Insulin Tolerance Test [10]

On weeks 7 and 8, every group was subjected to IPGTT and IPITT, respectively. IPGTT was performed in the morning with an intraperitoneal injection of 1 g/kg glucose after 12 h of fasting. Blood glucose levels were measured at 0, 15, 30, 60, and 120 min and the area under the curve (AUC) of blood glucose and time was calculated. IPITT was performed by injecting 1 U/kg insulin (Biosynthetic Human Insulin Injection, Novo Alle, DK 2880 Bagsvaerd, Denmark) intraperitoneally in mice following 4 h fasting. Blood glucose levels were subsequently determined at 0, 15, 30, 60, and 120 min and the results were processed. Blood glucose value at 0 min was considered as 100%, while the values obtained at 15, 30, 60, and 120 min were subtracted from the 0-min value. The absolute value was divided by the 0-min value, followed by its multiplication with 100 and the measurement of AUC of the value of the percentage versus time.

### 2.6. Reagent

Metformin hydrochloride tablets were purchased from Simo-American Shanghai Squibb Pharmaceuticals, Ltd. (H20023371). Antibodies to GAPDH, Akt, Glut4 and Anti-Rabbit IgG-HRP, and Anti-mouse IgG-HRP were purchased from Santa Cruz Biotechnology, Inc. The antibodies to INSR, IRS-1, and PI3K were purchased from Cell Signaling Technology, Inc. RIPA Lysis Buffer was purchased from Beyotime company, Cat. number P0013B; PVDF was purchased from Millipore, Cat. number IPVH00010.

### 2.7. Biochemical Analysis

At the end of week 9, mice were fasted for 12 h and blood samples were collected and immediately centrifuged (4°C, 1,500 ×g, 10 min) to obtain serum for the analysis of blood glucose, insulin, and glycated serum protein (GSP) levels. The homeostasis model assessment of insulin resistance (HOMA-IR) was calculated according to the following formula: HOMA-IR = fasting blood glucose (mmol/L) × fasting serum insulin (mIU/L)/22.5. Fasting insulin (FINS) levels were evaluated using an enzyme-linked immunosorbent assay (ELISA) kit (Cusabio Biotech Co., Ltd, Wuhan, China). The serum total cholesterol (TC), high-density lipoprotein cholesterol (HDLC), low-density lipoprotein cholesterol (LDLC), and triglyceride (TG) levels were measured using commercial kits (Nanjing Jiancheng Bioengineering Institute).

### 2.8. Hematoxylin-Eosin (H&E) Staining

Mice liver, pancreas, and skeletal muscle were fixed in 4% paraformaldehyde for 24 h, followed by paraffin embedding, tissue sectioning, and hematoxylin and eosin (H&E) staining. The morphologies and structures of these tissues were visualized and photographed using an optical microscope.

### 2.9. Western Blot Analysis

For the preparation of protein samples, 50 mg tissue sample from the skeletal muscle subjected to liquid nitrogen freezing was homogenized in radioimmune precipitation assay (RIPA) buffer and centrifuged for the separation of the supernatant and fragments. The protein concentration was determined using the bicinchoninic acid (BCA) protein assay. Equal amounts of protein samples were separated using sodium dodecyl sulfate polyacrylamide gel electrophoresis (SDS-PAGE) and transferred onto polyvinylidene fluoride (PVDF) membranes. The membranes were blocked with 5% nonfat milk for 1 h and overnight incubated with primary antibodies at 4°C. After incubation, the membranes were washed with TBS-T and incubated with horseradish peroxidase-conjugated secondary antibodies for 1 h. The membranes were washed with TBS-T and the protein bands detected with enhanced chemiluminescence reagent and Bio-Rad ChemiDoc™ XRS+. The integrated optical density was calculated using Quantity One.

### 2.10. Statistical Analysis

Statistical analysis was performed using SPSS (19.0 version). All data are shown as the mean ± standard error of the mean (SEM). One-way analysis of variance (ANOVA) was used for multigroup comparisons, followed by least significant difference (LSD) or Student-Newman-Keuls (SNK) test for the comparison between two groups. Differences were considered statistically significant at a *p* value of <0.05.

## 3. Results

### 3.1. Detection of the Major Components of SGY Preparation with HPLC Fingerprint System

**Figure 1:**
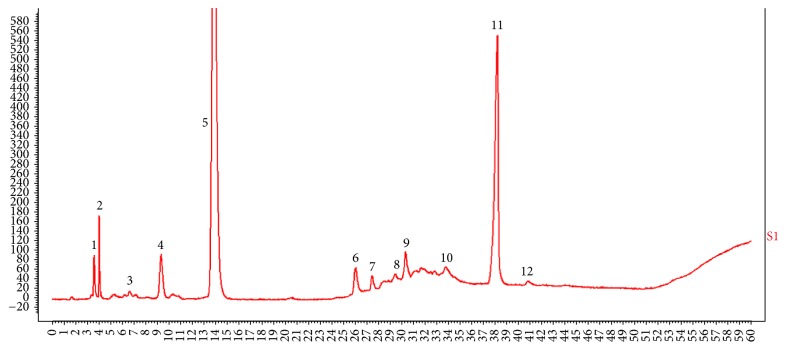
HPLC-ELSD fingerprint analysis of SGY preparation. In total 12 peaks were labeled; peak 1 is daidzein, peak 4 is daidzin, peak 5 is puerarin, peak 7 is momordicoside A, and peak 11 is 1-DNJ.

Reference chromatographic fingerprint for SGY preparation was generated based on 5 batches of samples. A good separation and reproducible chromatogram was achieved and 12 peaks were marked as the common peaks (from peak 1 to peak 12) (Figure 1). Our method determined their features, especially those identities of daidzein (peak 1), daidzin (peak 4), puerarin (peak 5), momordicoside A (peak 7), and 1-DNJ (peak 11); the similarities of the SGY preparation were calculated compared with the reference chromatogram; the least similarity value of these samples was 0.93, which indicated that the samples had similar chemical compositions and this reference chromatogram could be applied as a standard HPLC fingerprint.

### 3.2. Effects of SGY Preparation on PBG, BW, and GSP

**Figure 2:**
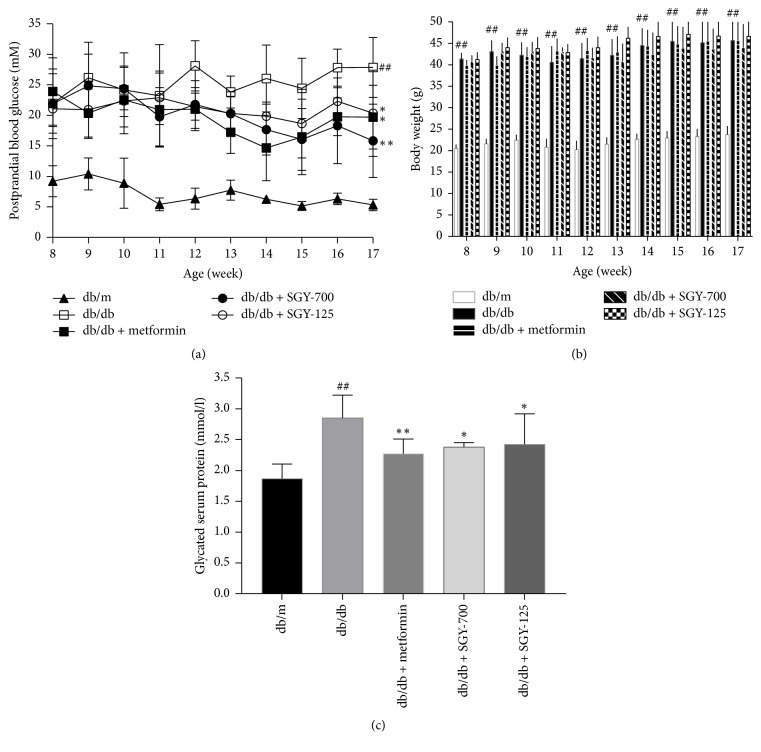
Effect of SGY preparation on postprandial blood glucose (PBG) (a), body weight (BW) (b), and glycated serum protein (GSP) (c). PBG and BW were determined from 14:00 to 14:30 on the first day of each week. GSP was measured at the end of week 9. ^##^*p* < 0.01 versus* db/m*; ^*∗*^*p* < 0.05, ^*∗∗*^*p* < 0.01 versus* db/db* mice treated with saline. Results are presented as means ± SEM (*n* = 6 in each group).

Postprandial blood glucose levels were significantly higher in* db/db* mice than in* db/m* mice. PBG level of the model group undulated during the experiment. In comparison with the model control group, metformin and SGY preparation groups showed a significant reduction in PBG level from week 6 (mice were 14-week old) (*p* < 0.05). At the end of the experiment,* db/db* mice were given a gavage of SGY preparation and showed 43.2% and 30.0% lower glucose levels than those of the groups given a gavage of saline (Figure 2(a)). However, no obvious differences were observed between BW of* db/db *mice treated with SGY preparation and the model controls (Figure 2(b)). We evaluated the effect of SGY preparation in* db/m* mice in our preexperiments and found no changes in PBG and BW of* db/m* mice treated with SGY preparation. As shown in Figure 2(b), GSP level was higher in the model group than in the* db/m* group (*p* < 0.01) and decreased significantly by 20.4%, 16.5%, and 15.1% following treatment with metformin, SGY-700, and SGY-125, respectively (*p* < 0.05).

### 3.3. Sangguayin Preparation Increases the Insulin Sensitivity in* db/db* Mice

**Figure 3:**
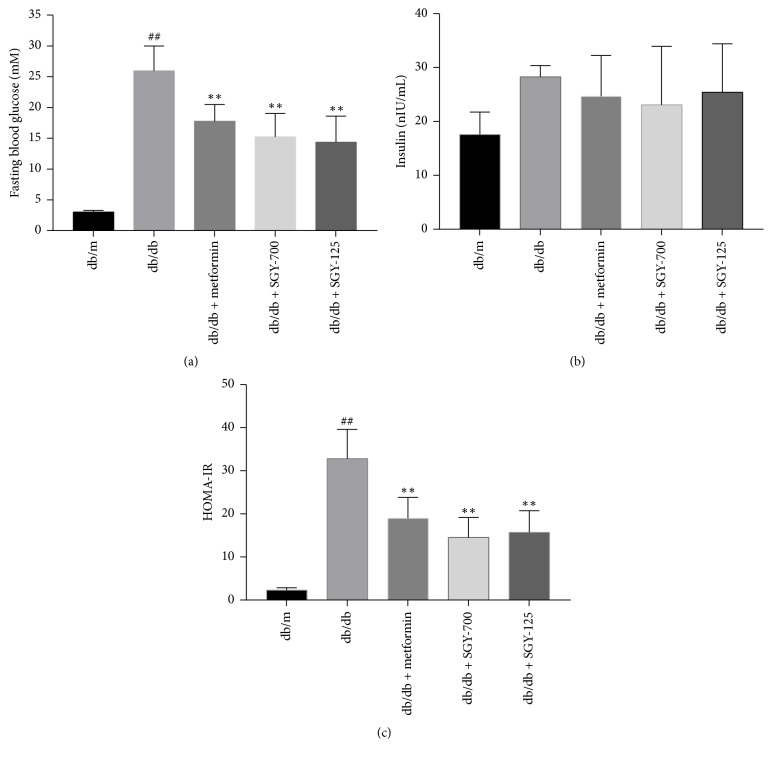
Effects of SGY preparation on FBG, FINS levels, and HOMA-IR. FBG (a), FINS (b), and HOMA-IR index (c) were measured in each group after 9 weeks of treatment. ^##^*p* < 0.01 versus* db/m*; ^*∗∗*^*p* < 0.01 versus* db/db *mice treated with saline. Results are presented as means ± SEM (*n* = 6 in each group).

As shown in Figure 3(a), the fasting blood glucose and serum insulin level of diabetic mice were higher than those of* db/m* mice and reduced following metformin and SGY preparation treatment (*p* < 0.01); however, the difference in the serum insulin concentration between groups was not significant. The results of HOMA-IR showed that the insulin sensitivity was severely affected in* db/db* mice, while the treatment with metformin and different doses of SGY preparation for 9 weeks significantly increased the insulin sensitivity in* db/db* mice.

### 3.4. Effects of SGY Preparation on IPGTT and IPITT in* db/db* Mice

**Figure 4:**
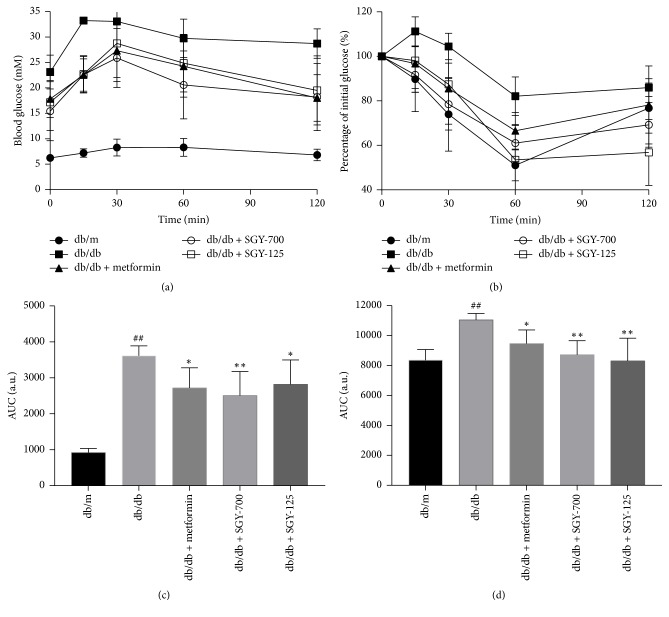
IPGTT (a) and IPITT (b) analysis and calculation of AUC for IPGTT (c) and IPITT (d). ^##^*p* < 0.01 versus* db/m* mice; ^*∗*^*p* < 0.05 and ^*∗∗*^*p* < 0.01 versus* db/db *mice treated with saline. Results are presented as means ± SEM (*n* = 5 in each group).

Type 2 diabetes mellitus is characterized by glucose intolerance and IR. We, therefore, performed the glucose tolerance and insulin tolerance tests at weeks 7 and 8. As shown in Figures 4(a) and 4(b), impaired glucose tolerance was observed in* db/db* mice, which was significantly improved by metformin and SGY preparation treatment. The AUC of the metformin and SGY preparation group was significantly lower than that of the model control. In addition, IR was observed in* db/db* mice, which was ameliorated by metformin and SGY preparation treatment (Figures 4(c) and 4(d)).

### 3.5. SGY Preparation Decreased the Serum Lipid Profile

**Figure 5:**
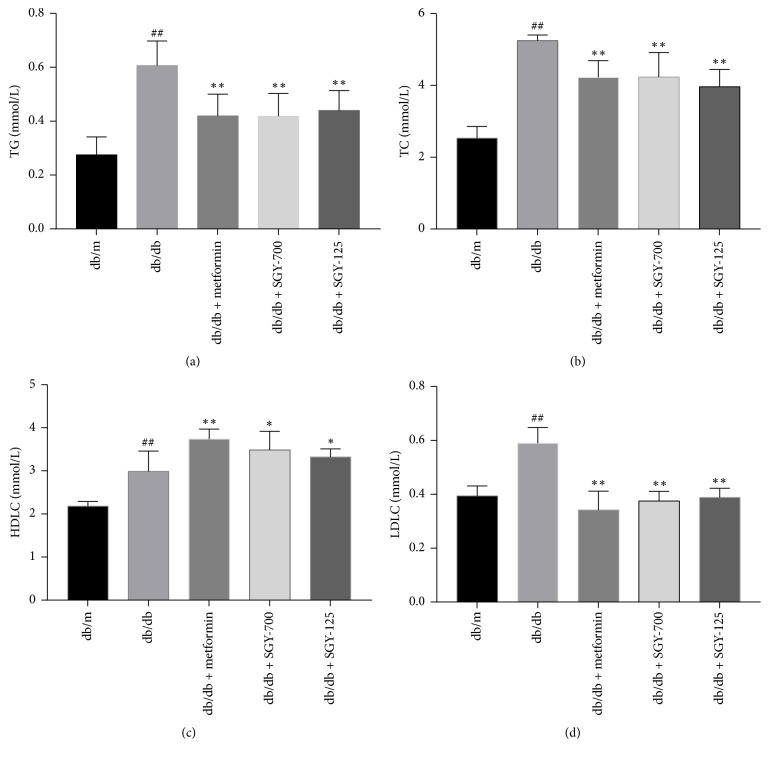
SGY preparation improved serum lipid levels in* db/db* mice. TG (a); TC (b); HDLC (c); and LDLC (d). ^##^*p* < 0.01 versus* db/m*; ^*∗*^*p* < 0.05 and ^*∗∗*^*p* < 0.01 versus* db/db *mice treated with saline. Results are presented as means ± SEM (*n* = 6 in each group).

The serum concentrations of TC, TG, LDLC, and HDLC were markedly increased in the model control group compared to in the* db/m* group (*p* < 0.01). SGY preparation and metformin treatment inhibited the increase in the levels of serum TC, TG, and LDLC and decreased the level of HDLC in the model control group (*p* < 0.01) (Figure 5). Thus, SGY preparation exhibited its beneficial effects by regulating serum lipid levels in* db/db* mice.

### 3.6. Effects of SGY Preparation on the Pathology of Pancreas, Liver, and Skeletal Muscle in* db/db* Mice

**Figure 6:**
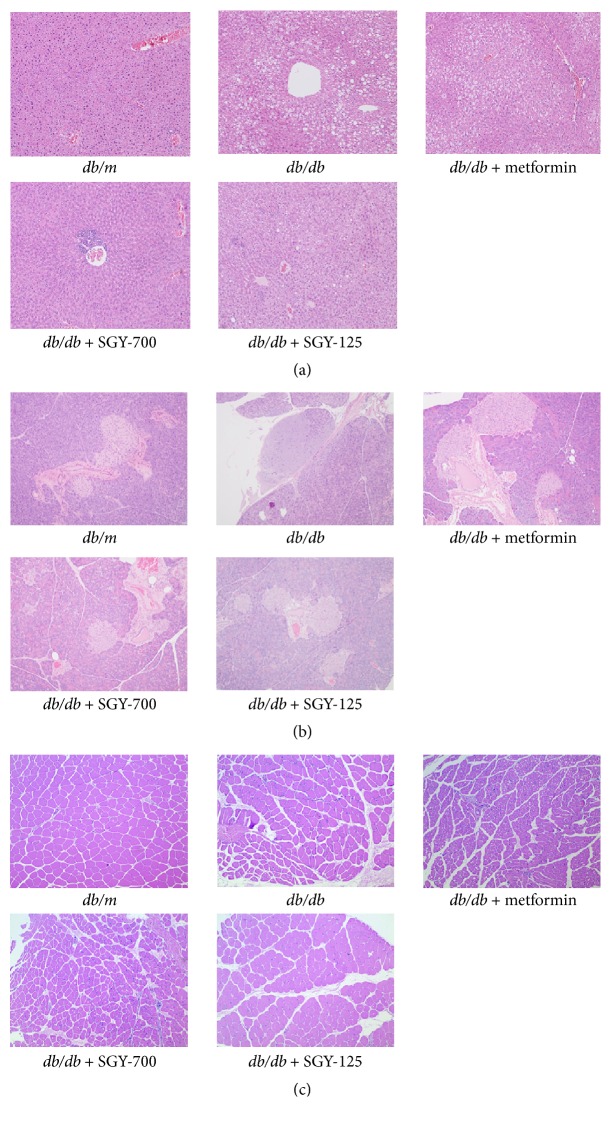
Effects of SGY preparation on the liver (a), pancreas (b), and skeletal muscle (c) of* db/db* mice, as assessed by H&E staining. Original magnifications of histological sections are ×100.

We performed histopathological examinations to confirm whether SGY preparation may improve hepatic steatosis and protect pancreatic islets and muscle fibers from deformation. As shown in Figure 6(a), model mice exhibited noticeable hepatic steatosis with respect to* db/m* mice. The lipid droplet accumulation was significantly reduced after the oral administration of metformin and SGY preparation for 9 weeks. As shown in Figure 6(b), the model mice had frequently atrophied pancreatic islets, while metformin and SGY preparation treatment reduced this effect.  Figure 6(c) demonstrates the deformation of muscle fibers in model mice; these pathological changes were partially improved by metformin and SGY preparation treatments.

### 3.7. Effects of SGY Preparation on the Expression of Proteins from Phosphatidylinositol-4,5-bisphosphate 3-kinase (PI3K)/Protein Kinase B (Akt) Signaling Pathway in the Skeletal Muscle

**Figure 7:**
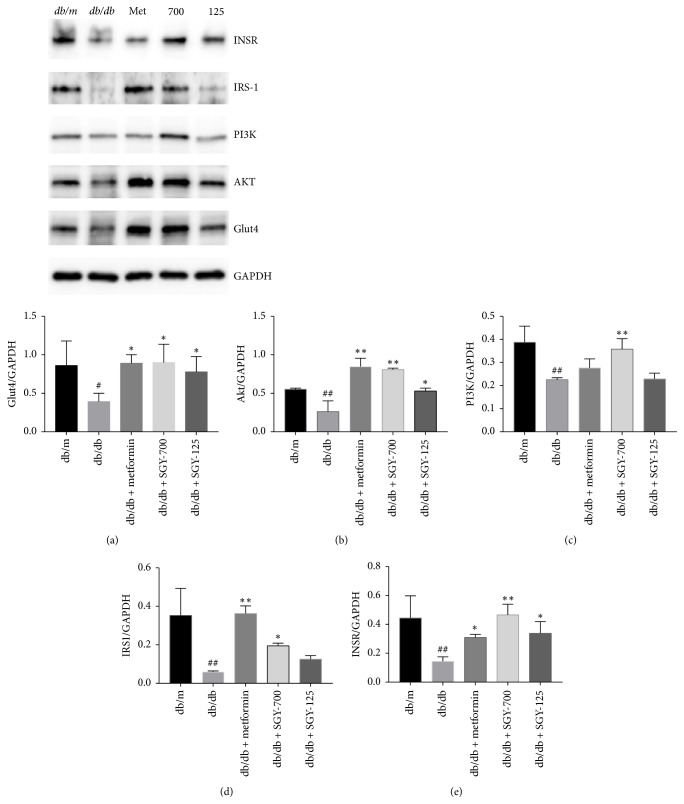
Effects of SGY preparation on PI3K/Akt pathway in the skeletal muscle of* db/db* mice, as evaluated by western blot analysis. Expressions of INSR, IRS-1, PI3K-p85, Akt, and GLUT4; ^#^*p* < 0.05 and ^##^*p* < 0.01 versus* db/m* mice; ^*∗*^*p* < 0.05 and ^*∗∗*^*p* < 0.01 versus* db/db *mice treated with saline. Results are presented as means ± SEM (*n* = 3 in each group).

The expression of the proteins such as insulin receptor (INSR), insulin receptor substrate 1 (IRS-1), PI3K, and Akt was significantly lower in the skeletal muscle of the model control group than in the* db/m* group. However, as shown in Figure 7 the oral administration of SGY preparation and metformin for 9 weeks significantly increased the expression level of these proteins. Moreover, GLUT4 expression was significantly higher in the groups administered with SGY preparation and metformin than in the model group. These results demonstrate that SGY preparation may regulate PI3K/Akt signaling pathway to ameliorate hyperglycemia in mice.

## 4. Discussion

In recent years, TCM is accepted as an efficacious remedy to prevent and treat various diseases. TCM has been used as a multiherbal formula to enhance its efficacy and/or reduce toxic effects. Many formulations have been proven effective for the treatment of diabetes [11, 12]. Given the complexity of T2DM pathogenesis and severe neopathy of multiple systems, no single drug component can be used to prevent or treat all events. Multiherbal formulations may serve as a promising strategy for the prevention and treatment of T2DM [13].

Sangguayin preparation is comprised of total alkaloids, saponins, flavonoids, and polysaccharides from* M. alba*,* M. charantia*,* P. thomsonii*, and* D. rhizoma*, respectively. The stable preparation process of the four effective fractions has been defined after long-term studies. In addition, the key ingredients of every effective fraction were determined and included 1-DNJ, momordicoside A, and puerarin. These fractions may serve as an integral contributing factor responsible for improving IR in animal models of T2DM. 1-DNJ, a key total alkaloid from* M. alba*, was recently identified as one of the principal antidiabetic constituents [14, 15]. Moreover, saponins such as momordicoside A are an active ingredient of* M. charantia* and known to reduce the lipid accumulation in 3T3-L1 cells and prevent IR [16, 17]. The flavonoids from* P. thomsonii* mainly contain puerarin, while the polysaccharides from* D. rhizoma* improve IR and prevent oxidative stress [18, 19].

In this study, we investigated the efficacy of SGY preparation in leptin receptor-mutant* (db/db)* mice—a spontaneous animal model of T2DM [20]. This mouse model presents syndromes, including hyperglycemia, obesity, and hyperlipidemia, while the disease pathogenesis and process are similar to human DM. In our study,* db/db* mice were diabetic and obese as compared with the normal control* (db/m)* group. SGY preparation effectively decreased the blood glucose level from week 4 (Figure 2) and its efficacy was comparable with that of the first-line oral antidiabetic drug, metformin; however, no significant differences in the dietary intake were observed as compared with* db/m* group. No significant change was observed between BW of mice treated with SGY preparation and the model group. Type 2 diabetes mellitus is associated with reduced glucose tolerance and IR [21]. We performed IPGTT and IPITT tests and found damaged glucose and insulin tolerance in the model group. On the other hand, the treatment of mice with SGY preparation ameliorated these effects (Figure 4) and alleviated the pathological changes in the liver, pancreas, and skeletal muscle tissues (Figure 6), indicative of the curative effect of SGY preparation in T2DM. Thus, the disturbance in the lipid metabolism induces the oversecretion of endocrine factors, which subsequently decrease the biological activity of insulin [22, 23]. Lipid metabolism disorder is mainly characterized by high levels of TG, TC, and LDLC and low levels of HDLC. Our results show that these abnormalities were partially ameliorated following treatment with SGY preparation and metformin for 9 weeks in* db/db* mice, but there was not a dose-dependent effect, even though different doses of SGY preparation were administered to the mice. The interaction among the complex ingredients of SGY preparation may be the reason for the above results. If the dose of the entire compound is increased, the positive actions of some ingredients may be offset by the others.

Insulin resistance in the skeletal muscle is considered as the main cause of T2DM [24]. Insulin stimulates the absorption of glucose by activating a series of signal transduction pathways following insulin binding. In this process, insulin exerts its physiological functions through the postreceptor cascade after binding to INSR on the surface of the membrane. INSR tyrosine kinase phosphorylates tyrosine residues on IRS proteins, which serve as docking proteins for downstream signaling molecules such as PI3K. PI3K is the key protein involved in glucose metabolism. The phosphorylated Akt protein increases following activation of PI3K and further activates GLUT4 [25]. Studies suggest that the clearance of circulating glucose in muscle cells depends on the increasing GLUT4-mediated glucose uptake [26–30]. Therefore, we evaluated the expressions of INSR, IRS1, PI3K, Akt, and GLUT4, the major proteins closely related to insulin resistance in the skeletal muscle.

## 5. Conclusions

In summary, the present study showed that SGY preparation may be used both as a food and medicine and reduces the blood glucose level, decreases lipid profiles, and ameliorates IR in* db/db* mice. The underlying molecular mechanism may be related to the regulation of PI3K/Akt signaling in the skeletal muscle. Our results suggest that SGY preparation may serve as a potential plant extract to alleviate metabolic syndrome.
